# Surgical referral coordination from a first-level hospital: a prospective case study from rural Nepal

**DOI:** 10.1186/s12913-017-2624-2

**Published:** 2017-09-25

**Authors:** Matthew Fleming, Caroline King, Sindhya Rajeev, Ashma Baruwal, Dan Schwarz, Ryan Schwarz, Nirajan Khadka, Sami Pande, Sumesh Khanal, Bibhav Acharya, Adia Benton, Selwyn O. Rogers, Maria Panizales, David Gyorki, Heather McGee, David Shaye, Duncan Maru

**Affiliations:** 1 Department of Surgery, Yale-New Haven Hospital, New Haven, CT USA; 2 Oregon Health & Sciences University, Portland, OR USA; 3 Bellevue Hospital Center, Ronald O. Perelman Department of Emergency Medicine, New York University School of Medicine, New York, NY USA; 4 Possible, Sanfebagar-10, Achham Nepal; 5 Department of Medicine, Division of Global Health Equity, Brigham and Women’s Hospital, Boston, MA USA; 6 Department of Medicine, Division of General Pediatrics, Boston Children’s Hospital, Boston, MA USA; 7 Department of Medicine, Division of General Internal Medicine, Massachusetts General Hospital, Boston, MA USA; 8 Department of Medicine, Harvard Medical School, Boston, MA USA; 9 United Nations Population Fund, Kathmandu, Nepal; 10 Institute of Medicine, Tribhuvan University, Kathmandu, Nepal; 11 Department of Psychiatry, University of California, San Francisco, San Francisco, CA USA; 12 Department of Anthropology, Northwestern University, Evanston, IL USA; 13 Program of African Studies, Northwestern University, Evanston, IL USA; 14 Biological Sciences Division, Department of Surgery, University of Chicago, Chicago, IL USA; 15 Vitals Global Healthcare, Floriana, Malta; 16 Division of Cancer Surgery, Peter MacCallum Cancer Centre, Melbourne, Australia; 17 Department of Surgery, University of Melbourne, Melbourne, Australia; 18 Department of Radiation Oncology, Icahn School of Medicine at Mount Sinai, New York, NY USA; 19 Massachusetts Eye and Ear Infirmary, Boston, MA USA; 20 Department of Otology and Laryngology, Harvard Medical School, Boston, MA USA; 21 Department of Global Health and Social Medicine, Harvard Medical School, Boston, MA USA

**Keywords:** Disease management, Case management, Referral and consultation, General surgery, Global health, Developing countries, Community health worker, Nepal

## Abstract

**Background:**

Patients in isolated rural communities typically lack access to surgical care. It is not feasible for most rural first-level hospitals to provide a full suite of surgical specialty services. Comprehensive surgical care thus depends on referral systems. There is minimal literature, however, on the functioning of such systems.

**Methods:**

We undertook a prospective case study of the referral and care coordination process for cardiac, orthopedic, plastic, gynecologic, and general surgical conditions at a district hospital in rural Nepal from 2012 to 2014. We assessed the referral process using the World Health Organization’s Health Systems Framework.

**Results:**

We followed the initial 292 patients referred for surgical services in the program. 152 patients (52%) received surgery and four (1%) suffered a complication (three deaths and one patient reported complication). The three most common types of surgery performed were: orthopedics (43%), general (32%), and plastics (10%). The average direct and indirect cost per patient referred, including food, transportation, lodging, medications, diagnostic examinations, treatments, and human resources was US$840, which was over 1.5 times the local district’s per capita income. We identified and mapped challenges according to the World Health Organization’s Health Systems Framework. Given the requirement of intensive human capital, poor quality control of surgical services, and the overall costs of the program, hospital leadership decided to terminate the referral coordination program and continue to build local surgical capacity.

**Conclusion:**

The results of our case study provide some context into the challenges of rural surgical referral systems. The high relative costs to the system and challenges in accountability rendered the program untenable for the implementing organization.

**Electronic supplementary material:**

The online version of this article (10.1186/s12913-017-2624-2) contains supplementary material, which is available to authorized users.

## Background

Worldwide, impoverished rural communities typically lack access to complex medical interventions [1, 2]. It is estimated that 11% of the world’s disability-adjusted life years can be attributed to surgically treatable disease [3], though this is likely an underestimate [4]. Rural populations typically receive surgical care within public district healthcare systems that have neither the capacity to effectively deliver surgical care within their district [5–7] nor to coordinate referral care outside their district [8]. Thus, rural patients do not receive much-needed surgical care.

While a growing literature has developed around the provision of essential surgical care in rural areas [9], there exist limited data describing the operations of rural surgical referral networks. Given the specialized nature of surgical care, the importance of high volumes for quality, and the challenges of getting trained surgical teams to rural areas, referral programs have to be part of the broader system of surgical access for rural populations. Additionally, surgical patients require a consistent point of contact for ongoing clinical management and need well-coordinated post-discharge care [10]. The lack of these referral networks leads to excess morbidity and mortality, and has encouraged short-term surgical “camps”, which have large variations in cost, quality, and safety [11–13].

Here, we describe an attempt at developing a surgical referral program at a government hospital in Achham District in the Far-Western Development Region of Nepal. The hilly terrain and limited road infrastructure are such that the hospital lies 14 h away by road from the nearest tertiary care center, and 30 h from the capital of Kathmandu where most specialty surgical care is centered. The hospital is managed by the non-profit organization *Possible* in a public-private partnership approach. The goal of the program was to improve access to surgical care within the public sector system by coordinating referrals.

In this prospective case study of the program, our primary goal was to evaluate program implementation, aiming to better understand program costs and operations within the World Health Organization (WHO) Health Systems Framework: governance, finance, human resources, medical products, information systems, and delivery systems [14]. Through this analysis, we hoped to better inform the challenges and pitfalls facing rural hospitals in developing surgical referral systems.

## Methods

### Setting

*Possible* and the Nepal Ministry of Health are engaged in a public-private partnership in Achham District in far west Nepal [15]. Although over 80% of Nepal’s nearly 30 million people live in rural regions [16], nearly all of advanced medical care, medical schools, and surgical capacity are clustered in the major cities [17]. Achham is itself one of the poorest districts in Nepal, located 12 h by uncertain roads from a commercial airport and 14 h from the nearest hospital with any sort of specialty surgical capacity. Based on human development estimates, Achham ranks 73rd out of 75 districts in Nepal and has a mean per capita income of $536 [18]. It was severely affected by the ten year civil war that ended in 2006 [19].

In this context *Possible*, a non-profit healthcare provider, manages the first-level government facility, Bayalpata Hospital, and frontline community health workers (CHWs) [20, 21]. At the time of the study, Bayalpata Hospital had 25 inpatient beds and treated over 40,000 outpatients annually. Similar to many rural areas, local surgical care was limited to basic surgical procedures that fall under the purview of a generalist physician: cesarean delivery, laparotomy, and open fracture treatment [22, 23].

For this prospective case study, we used patient chart reviews, cost data, and semi-structured key informant interviews to guide our analysis.

### Chart reviews

We conducted a prospective case study of patients referred for outside surgical care from June 1st, 2012 through April 6th, 2014. All patients included in the study were seen by clinicians at Bayalpata Hospital during the aforementioned dates. Inclusion criteria for the program were defined by an independent external funding mechanism and all patients meeting these criteria were included in this analysis [24–27]. Chart reviews were conducted (by author MF, SP, and NK) for all patients to collect descriptive data on the type and costs of surgery. All data were routine programmatic data collected within the scope of the hospital’s (Bayalpata Hospital) surgical program and de-identified for research analysis. Seven-day follow-up was defined as either an in-person visit to Bayalpata Hospital or a phone call from Community Health Program staff. This time frame was selected for logistical convenience for the follow-up program, given there was insufficient clinical capacity to make for more tailored, individualized follow-up timelines. Thirty-day follow-up was chosen based on standards for evaluating 30 day perioperative mortality [28] and was defined as a phone call with the patient or caretaker if the patient was a minor, an in-person visit with the Community Health Program and a clinician at Bayalpata Hospital, or a home visit with a CHW.

### Cost data

Given the lack of data on disability weighting and the breadth of the surgical program, we conducted a cost analysis. Costs were converted from Nepalese Rupees to United States Dollars at the 2014 exchange rate. Owing to the limited and focused nature of this study, we did not adjust for inflation or purchasing power parity, and did not do discounting. Both direct costs (medications, diagnostics, and treatments) and indirect costs (transportation, food, and lodging) were based upon bills provided per service by the referring hospitals and other vendors. Since the program did bulk purchasing, these costs are averaged over multiple patients.

### Key informant interviews

Author SK conducted open-ended, semi-structured key informant interviews with clinical and operational staff leadership (*n* = 10) to supplement the chart reviews. Providing feedback on programs is a routine part of *Possible’s* organizational culture. No personally identifiable data were collected during these interviews.

We employed a case study approach to guide our qualitative method design and we used an inductive thematic framework for qualitative data analysis of the key informant interviews (see Additional file 1 for the key informant interview guide) [29]. Responses were classified according to the WHO Health Systems Framework and sorted into respective categories. The authors (MF, CK, and DM) agreed upon the classification of responses within the Framework. This Framework was chosen because surgical referral systems need to be conceptualized within the broader healthcare system, particularly as we consider questions of scale, access, and quality. In addition, this Framework provides a multi-faceted interrogation of the healthcare delivery system that goes beyond the common focus areas of healthcare personnel and financing. In our inductive thematic framework, we applied open coding procedures to allow for themes to emerge from the data. Those themes were then grouped and checked against the original WHO Framework.

## Results

### Surgical referral and care coordination program

Patients who presented for care at Bayalpata Hospital were evaluated by medical staff. If, based on the diagnostic and clinical assessment available at the hospital, the medical staff deemed surgery or further evaluation for surgery was required, a Community Health Program staff member interviewed the patient to assess travel readiness, family support, and arranged transport to the appropriate referral center. A staff member then travelled with the patient and their caretaker to the referral center. Travel was arranged to fit the urgency of the patient’s condition and any other conflicting priorities (e.g. childcare or income generation). The staff member accompanied the patient and their family to the hospital and helped navigate, as needed, the referral hospital itself. This would include time awaiting the results of diagnostic tests necessary to confirm whether surgery was indicated. The staff member also arranged for housing and food at the referral hospital.

All logistical support was essential because many of the patients and their families were illiterate and of low socioeconomic position; without accompaniment, the patients were likely to be turned away or otherwise made to wait extensively at the hospital. In addition, many patients and families had never left Achham, and would have faced significant challenges in navigating the healthcare system and arranging logistical support like food and lodging. The primary goal of this process was to ensure that patients were supported throughout the referral and received surgery within a reasonable time frame.

After successful treatment and hospital discharge, the staff member accompanied the patient back to Bayalpata Hospital for follow-up. If necessary, staff coordinated follow-up appointments at specialist referral centers, including the logistics of travel, food, and lodging for patients and their caretakers. To the extent possible, basic follow-up for wound care, medication management education, and management of ongoing symptoms were managed locally by the Community Health Program staff and clinicians at Bayalpata Hospital. The external independent funding source coordinated by *Possible* covered all costs incurred in the operation of this referral program, including follow-up care.

**Fig. 1:**
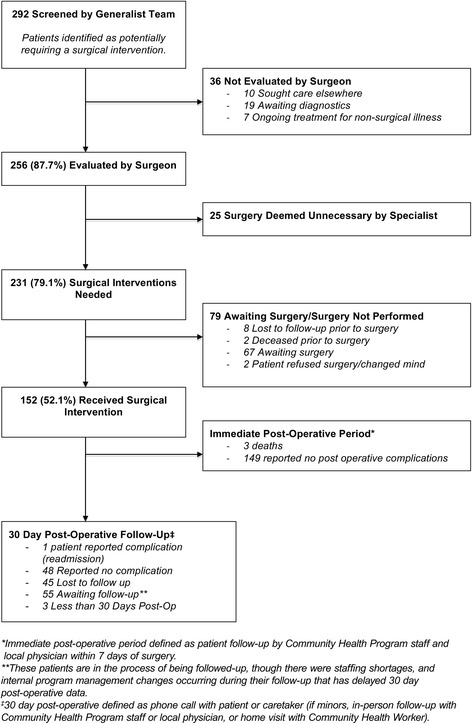
Study Flow Chart

**Fig. 2:**
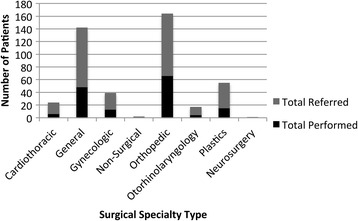
Total Referred and Performed Surgeries

Between June 1st, 2012 and April 6th, 2014, a total of 83,891 patients were seen at the Bayalpata Hospital outpatient department and 7503 were seen in the emergency department. Of these patients, 292 (0.3%) patients were identified as possible surgical candidates. Of those, 152 (52%) underwent surgery. Among those who received surgery, 149 (98%) had successful surgery without complication up to seven days post-operatively and three (1%) died post-operatively (See Fig. 1). Figure 2 provides a breakdown of surgeries by specialty. Total costs of the case study are as follows: $46,200 for food, transportation, and lodging; $75,500 for medications, diagnostics, and treatments; and $38,500 for human resources. The average cost per patient referred was approximately $840, exceeding the Achham District mean per capita income of $536 [21].

### Implementation challenges

We organized the data from the key informant interviews to map the components of the program using the WHO Health Systems Framework. This provided a structured assessment of strengths and challenges in implementation of the surgical care coordination program. Table 1 illustrates the intervention components of this surgical referral coordination program using WHO Health Systems Framework blocks. Although our experience demonstrated that this program was feasible, because of the challenges discussed here, particularly around governance, finance, and service delivery systems, the program was ultimately terminated as *Possible* and its government partners decided to focus scarce resources on building local capacity rather than referral systems.

**Table 1:** Understanding surgical triage and referral intervention within the WHO Health Systems Framework

Blocks 1 & 2: Governance & Finance
a) Public sector insurance scheme covering referral care for all patients eliminates need for independent funding mechanism (includes travel, food and lodging, and free treatment for all patients in the program).
b) Logistics partnerships, such as lodging in Kathmandu, have greatly improved patient comfort during long treatments far from home.
c) Partnerships with care providers, such as an orthopedic rehabilitation hospital, provide services otherwise unavailable locally.
d) A regulatory framework, likely embedded within financing system, is required to hold providers accountable to quality, access, and safety.
Blocks 3: Medical Products
a) Need for improved supply chain around essential surgical triage and diagnostics, including blood products, x-ray, ultrasound, splinting, and advanced imaging. Yet at a local level, human resources are more fundamental bottlenecks.
Block 4: Human Resources
a) Partner physicians at private and academic centers provide phone and telemedicine consultations to physicians.
b) Staff training on cases meeting criteria for funding to encourage active case finding.
c) Staff training on the diagnostic and therapeutic options, and limitations at referral sites.
d) Staff training on pre-surgical referral patient management and triage.
e) Staff training on diagnosis of commonly referred surgical diseases (e.g. fracture, rheumatic heart disease, and osteomyelitis).
f) Visiting surgical teams for on-site training and local co-management of cases.
Block 5: Information Systems
a) Staff maintain a follow-up registry that automatically alerts staff regarding whom and when to follow-up.
b) Staff maintain an up to date contact list for patients, referral care providers, support staff, CHWs, and partner organizations providing logistics support.
c) Ultimately, an integrated electronic health record is required, and the Possible team has deployed this following the study.
Block 6: Delivery Systems
a) First level hospital Community Health Program as “focal point” in coordinating referral surgical care.
b) Active case finding in the community through coordination with CHWs.
c) Frequent phone communication between patients, families, and staff and home visits by CHWs.
d) In hospital follow-up of all referred patients by staff and clinical staff at one month post-surgery.
e) Part time staff at referral center assures timely provision of care and further coordinates with local staff.
f) Subsidized transportation to and from referral center (often >12 h by bus each way) and transport for follow-up care.
g) Emailed and phone conversations between staff at local and referral sites of care for coordination.
h) Use of performance metrics including follow-up rates and complications.
i) Community Health Program staff coordinate with local clinicians, patients, and referral care centers.
j) Staff accompany patients and help navigate distant medical centers assuring proper care is received.
k) Staff coordinate follow-up with referral centers and patients, organizing travel and other logistics.
l) Focus of referral relationships on collaboration with local teams and local staff education.
m) Hospital staff work closely with CHWs for follow-up and patient education.
m) Clinical staff provide patient education specific to condition and where patient is in referral care loop.
o) CHWs provide emotional support and can help with referral process if complication occurs.

## Discussion

### Blocks 1 & 2: Governance & Finance

A central challenge the program faced was that of limited national regulations around the costs, indications, and quality of surgical care. Governance and finance issues are fundamentally intertwined. In Nepal, certain primary care services and medicines are provided free of cost at government facilities. Since public sector services are frequently not available or considered to be of low quality, over 60% of healthcare expenditures in Nepal are paid for by patients at the point of care [30]. This number is likely significantly higher for surgical care, since, outside of cesarean sections, surgical care (even in the government system) levies fees to patients. A core constraint in surgical care delivery is thus the lack of a public sector financing regime. Along with that, there is no regulatory body that oversees the quality or standards of surgical care.

Referral centers charged surgical fees without appropriate justification or consideration of the patient’s socioeconomic position. Price negotiation was difficult due to a relative monopoly on surgical care by a small set of overburdened providers and a lack of an effective system for providing charity care.

### Block 3: Medical products

At the referral centers, medical products for safe surgery were generally available, owing to Nepal’s ability to import products from its open border with India. At a local level, limited diagnostic capacity made it challenging to accurately assess disease severity and determine the need for surgical intervention. It was difficult for clinicians to weigh the very real costs and dangers of an often treacherous 14 h jeep ride in highly uncertain clinical diagnostic scenarios posed by a lack of tests. Core triage and stabilization products like blood products, opioid analgesics, ultrasound, x-ray, splinting and casting, and intubation equipment were available. Yet skilled staff comfortable in assessment and stabilization were often unavailable.

### Block 4: Human resources

At the time of this study, there were no full time public sector general surgeons, orthopedic surgeons, or obstetricians in the entire 2.5 million population of far west Nepal. Nearly all surgeons in Nepal were based in one of the few urban centers of the country. Key informants highlighted the need for further staff training on the indications and use of certain services or products (e.g. blood transfusion, opioid pain relievers, and skills training on basic echocardiography for assessment of rheumatic heart disease). The team has trialed visiting clinicians from Kathmandu and internationally to lead training sessions on ultrasound and pain management, and further workshops will be developed as the need arises. Key informants noted that electronic consultations with specialist physicians in Kathmandu have further improved patient management for conditions such as burns and fractures, though urgent consultations remain difficult to obtain.

### Block 5: Information systems

Previous information technology systems in place at Bayalpata Hospital did not smoothly facilitate follow-up of post-surgical patients. Subsequently, Community Health Program staff designed a Microsoft Access™ database that automatically alerted them to follow-up with a specific patient at specific points within that patient’s referral cycle. This system facilitated more efficient allocation of limited staff time and ensured patients were not lost to follow-up. Data management skills and computer literacy were also identified as areas necessitating further refinement among key programmatic stakeholders. As a result of this and other programmatic challenges, *Possible* ultimately decided to implement an integrated electronic health record and continuous surveillance system appropriate for the resource limitations of Achham [31, 32]. This went live February 5th, 2015, after study completion.

### Block 6: Service delivery systems

Early in the program’s implementation, Community Health Program staff travelled 14 h one way with patients several times per week, sometimes travelling even farther to Nepal’s capital, Kathmandu. This travel burden added new costs and unnecessary stress to patients and staff alike. Consequently, *Possible* hired a fulltime member based at the primary referral center to facilitate transfer of patients and to lessen the travel burden on staff. This new staff member streamlined communication between the local provider and referral provider. Previously this had been inconsistent and the revised structure served to improve point of care patient advocacy in the main referral hospital. Additionally, group transport of patients, caretakers, and *Possible* staff members were coordinated when possible to reduce individual staff travel burden.

Community Health Workers were the foundation of the follow-up system and were critical to maintaining open lines of communication between the community and the healthcare system. CHWs responsible for patients within their villages relayed messages and physically located patients who may have lived several days walk away from Bayalpata Hospital, encouraging patients to seek initial clinical evaluation or follow-up care. The program had success in locating and following up patients in areas with highly engaged CHWs.

One key aspect of referral surgery is securing dignified, safe lodging for patients and their family members at the referral sites. Many families had never travelled to large urban areas, were unfamiliar with how to navigate hospitals and lodging requirements, and may have decided not to seek treatment because of fear of the unknown. To address this, *Possible* partnered with two organizations that specialized in providing lodging and logistical help to patients in Kathmandu and Nepalgunj, where the major referral centers were located. These collaborations greatly improved patients’ experience with referral care and lightened *Possible* staff burden while accompanying patients.

Families often were reluctant to engage in treatment because of the opportunity cost of time lost while receiving care. 57% of households in Nepal have at least one family member who has migrated in the past 10 years [21]. In Achham, where men are typically working outside the district, women are often faced with significant economic loss should they choose to travel to a referral center with a sick child instead of staying at home to care for their land and animals [33]. One patient, after being diagnosed with a large inguinal hernia, refused treatment because it was the harvest season and the risk of losing food and income was not worth the benefit of free medical care. Another patient’s mother insisted on taking her child back to Achham from Kathmandu before treatment was complete because her husband was left alone caring for their other children.

## Conclusions

We followed 292 surgical referral patients as part of a prospective case study on a surgical referral program at a district hospital in rural Nepal. While the total cost per patient—US$840—exceeded the local per capita income and led to discontinuation of the program, our case study reveals implementation strengths and challenges for consideration by other policymakers and program implementers.

Following the completion of this study, further growth of the *Possible* team led to a shift in funding and operational priorities towards expanding on-site surgical capacity rather than deepening referral care networks. Notably, the *Possible* team felt that the logistical complexities of the program did not justify the high costs, with per patient costs exceeding the local per capita income. As such, the funding of and operational support for referral surgeries ceased. The lessons learned here, both from costing and from operations, are relevant especially as countries like Nepal consider expanded health insurance packages that include more complex referral interventions. Critical to the durability of these interventions is integration within public sector systems and financing to both referral teams and receiving hospitals by some form of insurance. Nepal is indeed in the middle of deploying a national health insurance system precisely for services like advanced surgery.

The limitations of this study include a small sample size, a single study site for enrollment, limited enrollment criteria, and a lack of patient interviews and long-term follow-up data. The program itself was unique, both in its scope and in its being conducted within a public-private partnership between a non-profit organization and the government. These all limit the generalizability of our results to other settings. Additionally, we do not know whether most complications occurred prior to seven days post-operatively or were not captured in this metric. Furthermore, we were unable to perform a more rigorous patient outcomes and cost effectiveness assessment from a disability perspective.

The primary focus of the paper was to better explore the issues and operational challenges of rural surgical referral systems. Within that limited focus, the study achieved the primary goal of bringing out some of the major themes and challenges faced by teams such as those led by *Possible*. The experiences of this surgical referral coordination program suggest that, with sufficient managerial and financial inputs, effective surgical care can be achieved for even the most remote patients. Critical to meeting the challenge is encouraging dedicated staff members to continuously advocate for patients and their families, and establishing trustworthy partnerships at distant referral sites. A fundamental tension remains the extent to which local surgical capacity can be financed, capacitated, and expanded, both to reduce the high number of referrals and to improve the quality and oversight of those referrals.

## Additional file

Additional file 1: Key Informant Interview Guide. (DOCX 13 kb)

## Abbreviations

CHW: Community Health Worker, known locally in Nepal as Female Community Health Volunteers
WHO: World Health Organization
