# Supplementary material for: Surgical referral coordination from a first-level hospital: a prospective case study from rural Nepal
Source: BMC Health Serv Res. 2017 Sep 25;17:676. doi: 10.1186/s12913-017-2624-2 (PMC5613391; doi:10.1186/s12913-017-2624-2)
Supplement: Additional file 1: — Key Informant Interview Guide. (DOCX 13 kb) [file 12913_2017_2624_MOESM1_ESM.docx]

**Key-Informant Interview Guide**

Topics to focus on for interviews:

- Overall perceptions of safety
- Frequency of events reported
- Supervisor/manager expectations and actions promoting safety
- Teamwork within hospital
- Feedback and communication about errors

Questions to ask for interviews:

- Is patient safety ever put at risk to get more work done?
- Are there procedures for preventing errors from happening?
- What safety issues have come up in the past?
- Does your supervisor/manager seriously consider suggestions for improving patient safety?
- What are you actively doing to improve patient safety?
- Have mistakes led to positive changes around here?
- What do you do to evaluate the effectiveness of changes?
- Do people help each other out? For example, when one area of the hospital becomes very busy?
- Do you know how to report patient safety issues?
- How often do you speak up regarding issues endangering patient safety?
- Do you feel comfortable raising concerns to supervisors/managers regarding their decisions?
- Do you feel like your mistakes will be held against you?
